# Inhaled aviptadil for the possible treatment of COVID-19 in patients at high risk for ARDS: study protocol for a randomized, placebo-controlled, and multicenter trial

**DOI:** 10.1186/s13063-022-06723-w

**Published:** 2022-09-20

**Authors:** Maria Boesing, Kristin Abig, Michael Brändle, Martin Brutsche, Emanuel Burri, Björn C. Frye, Stéphanie Giezendanner, Jan C. Grutters, Philippe Haas, Justian Heisler, Fabienne Jaun, Anne B. Leuppi-Taegtmeyer, Giorgia Lüthi-Corridori, Joachim Müller-Quernheim, Reto Nüesch, Wolfgang Pohl, Frank Rassouli, Jörg D. Leuppi

**Affiliations:** 1 University Clinic of Medicine, Cantonal Hospital Baselland, Rheinstrasse 26, CH-4410 Liestal, Switzerland; 2 Faculty of Medicine, University of Basel, Klingelbergstrasse 61, CH-4056 Basel, Switzerland; 3 Cantonal Hospital St. Gallen, Rorschacherstrasse 95, CH-9007 St. Gallen, Switzerland; 4 Department of Pneumology, Medical Center University of Freiburg, Kilianstrasse 5, 79106 Freiburg, Germany; 5 St. Antonius Hospital Nieuwegein, Koekoekslaan 1, NL-3435 Nieuwegein, Netherlands; 6 Division of Heart & Lungs, University Medical Center Utrecht, Heidelberglaan 100, NL-3584 Utrecht, Netherlands; 7 AdVita Lifescience GmbH, Alte Bundesstrasse 20, 79194 Gundelfingen, Germany; 8 Department of Clinical Pharmacology and Toxicology, University Hospital Basel, University of Basel, Basel, Switzerland; 9 Hospital Schwyz, Waldeggstrasse 10, CH-6430 Schwyz, Switzerland; 10 Karl Landsteiner Institute for Clinical and Experimental Pneumology, Clinic Hietzing, Wolkersbergenstrasse 1, A-1130 Vienna, Austria

**Keywords:** Aviptadil, VIP, COVID-19, ARDS, SARS-CoV-2

## Abstract

**Background:**

Despite the fast establishment of new therapeutic agents in the management of COVID-19 and large-scale vaccination campaigns since the beginning of the SARS-CoV-2 pandemic in early 2020, severe disease courses still represent a threat, especially to patients with risk factors. This indicates the need for alternative strategies to prevent respiratory complications like acute respiratory distress syndrome (ARDS) associated with COVID-19. Aviptadil, a synthetic form of human vasoactive intestinal peptide, might be beneficial for COVID-19 patients at high risk of developing ARDS because of its ability to influence the regulation of exaggerated pro-inflammatory proteins and orchestrate the lung homeostasis. Aviptadil has recently been shown to considerably improve the prognosis of ARDS in COVID-19 when applied intravenously. An inhaled application of aviptadil has the advantages of achieving a higher concentration in the lung tissue, fast onset of activity, avoiding the hepatic first-pass metabolism, and the reduction of adverse effects. The overall objective of this project is to assess the efficacy and safety of inhaled aviptadil in patients hospitalized for COVID-19 at high risk of developing ARDS.

**Methods:**

This multicenter, placebo-controlled, double-blinded, randomized trial with 132 adult patients hospitalized for COVID-19 and at high risk for ARDS (adapted early acute lung injury score ≥ 2 points) is conducted in five public hospitals in Europe. Key exclusion criteria are mechanical ventilation at baseline, need for intensive care at baseline, and severe hemodynamic instability. Patients are randomly allocated to either inhale 67 μg aviptadil or normal saline (three times a day for 10 days), in addition to standard care, stratified by center. The primary endpoint is time from hospitalization to clinical improvement, defined as either hospital discharge, or improvement of at least two levels on the nine-level scale for clinical status suggested by the World Health Organization.

**Discussion:**

Treatment strategies for COVID-19 are still limited. In the context of upcoming new variants of SARS-CoV-2 and possible inefficacy of the available vaccines and antibody therapies, the investigation of alternative therapy options plays a crucial role in decreasing associated mortality and improving prognosis. Due to its unique immunomodulating properties also targeting the SARS-CoV-2 pathways, inhaled aviptadil may have the potential to prevent ARDS in COVID-19.

**Trial registration:**

ClinicalTrials.gov, NCT04536350. Registered 02 September 2020.

**Supplementary Information:**

The online version contains supplementary material available at 10.1186/s13063-022-06723-w.

## Administrative information

Note: the numbers in curly brackets in this protocol refer to SPIRIT checklist item numbers. The order of the items has been modified to group similar items (see http://www.equator-network.org/reporting-guidelines/spirit-2013-statement-defining-standard-protocol-items-for-clinical-trials/).

**Table:** 

Title {1}	Inhaled Aviptadil for the possible treatment of COVID-19 in patients at high risk for ARDS: A randomized, placebo-controlled, and multicentre trial.
Trial registration {2a and 2b}.	ClinicalTrials.gov, NCT04536350 Registered on 2^nd^ September 2020 https://clinicaltrials.gov/ct2/show/NCT04536350
Protocol version {3}	30.06.2022, Version 8
Funding {4}	This trial is financed by AdVita Lifescience GmbH, Gundelfingen, Germany. Co-Investigator Maria Boesing receives a personalized research grant within the program “Young talents in clinical research” from the Swiss Academy of Medical Sciences.
Author details {5a}	**Maria Boesing**, University Clinic of Medicine, Cantonal Hospital Baselland and Faculty of Medicine, University of Basel **Kristin Abig**, University Clinic of Medicine, Cantonal Hospital Baselland **Michael Brändle**, Cantonal Hospital St. Gallen **Martin Brutsche**, Cantonal Hospital St. Gallen **Emanuel Burri**, University Clinic of Medicine, Cantonal Hospital Baselland **Björn C. Frye**, Department of Pneumology, Medical Center University of Freiburg **Stéphanie Giezendanner**, University Clinic of Medicine, Cantonal Hospital Baselland **Jan C. Grutters**, St. Antonius Hospital Nieuwegein and Division of Heart & Lungs, University Medical Center Utrecht **Philippe Haas**, AdVita Lifescience GmbH **Justian Heisler**, University Clinic of Medicine, Cantonal Hospital Baselland and Faculty of Medicine, University of Basel **Fabienne Jaun**, University Clinic of Medicine, Cantonal Hospital Baselland **Anne B. Leuppi-Taegtmeyer**, Department of Clinical Pharmacology and Toxicology, University Hospital Basel and Cantonal Hospital Baselland **Giorgia Lüthi-Corridori**, University Clinic of Medicine, Cantonal Hospital Baselland and Faculty of Medicine, University of Basel **Joachim Müller-Quernheim**, Department of Pneumology, Medical Center University of Freiburg **Reto Nüesch**, Hospital Schwyz **Wolfgang Pohl**, Karl Landsteiner Institute for Clinical and Experimental Pneumology, Clinic Hietzing **Frank Rassouli**, Cantonal Hospital St. Gallen, **Jörg D. Leuppi**, University Clinic of Medicine, Cantonal Hospital Baselland and Faculty of Medicine, University of Basel
Name and contact information for the trial sponsor {5b}	Prof. Jörg D. Leuppi, MD, PhD Clinical Professor of Internal Medicine, University of Basel Head of the University Clinic of Medicine Cantonal Hospital Baselland Rheinstrasse 26 CH-4410 Liestal Phone: +41-61-925-21-80 E-Mail: joerg.leuppi@ksbl.ch
Role of sponsor {5c}	Professor Jörg D. Leuppi and his research team composed this study protocol together with collaborating partners. The study is conducted under the supervision of Jörg D. Leuppi. Jörg D. Leuppi and his research team are responsible for all submissions to relevant local authorities in order to obtain study approval. Prof. Leuppi is involved and has ultimate authority in every step of this study, including data collection, management, and analysis, interpretation of results, as well as composition of scientific reports and their submission for publication. AdVita Lifescience GmbH provides financial means for the conduction of this trial. Associates of AdVita contribute their experience with the investigational drug and its inhaled application.

## Introduction

### Background and rationale {6a}

The world has been experiencing an exceptional state, due to the SARS-CoV-2 pandemic. In the beginning of 2020, about 20% of individuals with the SARS-CoV-2 associated corona virus disease (COVID-19) suffered from a severe course, characterized by significant respiratory symptoms including the potentially lethal acute respiratory distress syndrome (ARDS) [1]. Since then, numerous studies have been initiated in order to evaluate therapeutic agents for the treatment of COVID-19. To date, an improved outcome in hospitalized patients has been shown in randomized controlled trials for anti-cytokine monoclonal antibodies (tocilizumab), Janus kinase inhibitors (baricitinib), and dexamethasone [2–4], which is reflected in current treatment recommendations by national and international health organizations and societies [5–8].

Further available therapy options for early outpatient treatment in high-risk patients include the monoclonal antibodies sotrovimab and bebtelovimab, antiviral substances remdesivir, nirmatrelvir/ritonavir, and molnupiravir, as well as inhaled corticosteroid budesonide and serotonin reuptake inhibitor fluvoxamine [9–14]. The monoclonal antibody combination casirivimab/imdevimab had been effective in reducing hospitalization risk and mortality till late 2021 but proved to have no benefit for the treatment of the emerging omicron variant [15]. At the time of setting up this study, other approaches like convalescent plasma, further antiviral antibodies and other experimental substances are being investigated [3, 16–18].

Despite the fast establishment and adaptation of recommendations for the clinical management of COVID-19, as well as large-scale vaccination campaigns, severe disease-courses still represent a threat, especially to patients with risk factors such as old age, arterial hypertension, or diabetes mellitus [19]. This indicates the need for alternative strategies to prevent and ameliorate respiratory complications associated with COVID-19, in order to effectively prevent intensive care (ICU) admissions and reduce mortality.

The results of the RECOVERY trial indicate that an excessive inflammatory reaction plays an important role in the pathophysiology of severe COVID-19 and the progression to ARDS [2]. In fact, severe cases of COVID-19 are associated with elevated serum levels of pro-inflammatory mediators [1, 20, 21]. SARS-CoV-2 specifically targets the surfactant-producing pulmonary alveolar type II (ATII) cells by binding to their angiotensin converting enzyme 2 (ACE2-) receptors and entering the cell [22]. Viral replication and infection of adjacent ATII cells then lead to a massive cytokine release and, consequently, to apoptosis and a critical decrease of surfactant production, which disrupts the alveolar gas exchange resulting in ARDS [22, 23].

Vasoactive intestinal peptide (VIP), a gut peptide hormone containing 28-residue amino acid peptides, was discovered and first synthesized in the seventies [24–26]. Next to fulfilling various effects in the nervous, digestive, cardiovascular, respiratory, and reproductive systems, VIP is physiologically highly localized in the lungs [27, 28]. There, it binds with ATII cells via the VPAC1 receptor, the same cell type to which the SARS-CoV-2 virus binds via the ACE2-receptor [22, 27]. When VIP binds to the ATII-cells, it inhibits NMDA-induced caspase-3 activity inside the cell, which in turn decreases production of the pro-inflammatory cytokines interleukin-6 and TNF-alpha [29–33]. It has been proposed as a modulator of lung inflammation and airway constriction [25, 26, 34], and its protective effects on pulmonary tissue have been shown in numerous animal models of lung injury in rats, guinea pigs, dogs, and sheep [35–37]. VIP was also shown to increase surfactant production by upregulation of choline phosphate cytidylyltransferase and C-Fos protein expression in ATII cells [38–40]. Recent data demonstrated that plasma levels of VIP are higher in patients with severe COVID-19, compared to healthy individuals and those with mild COVID-19 [41, 42], and that VIP can block SARS-CoV-2 virus replication in vitro [32].

Aviptadil, a synthetic form of VIP, might prevent COVID-19 patients from developing ARDS due to the above described anti-inflammatory properties. The presumed primary therapeutic mechanism of action of inhaled aviptadil is a combination of anti-inflammatory properties and induction of tolerogenic immune response of immune cells localized in the lungs [43, 44]. It has been shown to reduce interferon producing T cells, to dampen Th17-T-cells and to promote regulatory T-cells [43, 45, 46]. These immune-dampening effects have been previously described to occur in the alveolar compartment of sarcoidosis patients after inhalation of aviptadil, which demonstrates that its local application by inhalation is feasible and results in relevant immunological changes [43]. There is further promising evidence from a case report of a patient with pneumonitis resulting from check-point-inhibitor therapy for melanoma, in which the administration of inhaled aviptadil was well tolerated and led to dampening of alveolar inflammation, radiological and clinical improvement [47].

To date, there is no clinical evidence for the efficacy of inhaled aviptadil in COVID-19. However, it was recently observed in vitro that VIP can inhibit SARS-CoV-2 replication and reduce cellular proinflammatory cytokine production [32]. Two phase II trials have been announced recently on the US National Library of Medicine platform “ClinicalTrials.gov” (COVID-AIV (NCT04311697), AVICOVID-2 (NCT04360096)), which investigate aviptadil in patients with COVID-19 in the USA. Preliminary results of the COVID-AIV trial, which has been recruiting patients with COVID-19 and respiratory failure, indicate a promising antiviral effect of the intravenous administration of aviptadil [42]. In contrast, this current trial investigates the inhaled application of aviptadil in an earlier stage of disease, in patients at increased risk for ARDS. Inhaled aviptadil likely circumvents several potential side effects, like hypotension and tachycardia [43, 47].

Daily doses of up to 300 μg inhaled aviptadil have been shown to be safe in phase II trials for the treatment of sarcoidosis and pulmonary hypertension, as well as in a recently published phase I trial in the treatment of ARDS [43, 48–50]. Aviptadil has been given Orphan Drug Designation in the European Union and the USA for the treatment of ARDS and the inhaled application has been observed to be safe without severe side effects [43, 47, 51].

### Objectives {7}

The primary objective of this trial is to investigate the efficacy and safety of inhaled aviptadil in hospitalized COVID-19 patients at high risk for developing ARDS. The study will assess whether patients with COVID-19 under high risk for developing ARDS recover faster when they receive inhaled aviptadil in addition to standard care, compared to patients receiving standard care only. A secondary objective is the investigation of the overall course of disease under inhaled aviptadil in terms of need for mechanical ventilation, time requiring oxygen supplementation, infection-related biomarkers, and subjective severity of symptoms. The safety objective is to assess any potential harm of inhaled aviptadil.

### Trial design {8}

This study is a multicenter, placebo-controlled, double-blinded, randomized phase II trial with 132 adult patients. In a parallel group design, patients will be randomly allocated in a 1:1 ratio to inhale either aviptadil (67 μg three times a day) or normal saline (1 ml three times a day) for 10 days, or until hospital discharge, in addition to standard care. The study is conducted as a superiority trial.

### Public and patient involvement in the design of this protocol

Due to the novelty of the disease under investigation and the prevalent time pressure in the design phase of the trial, patient and public involvement in the design of this protocol was not applied.

## Methods: participants, interventions, and outcomes

### Study setting {9}

Recruitment of hospitalized patients takes place at the Cantonal Hospitals of Liestal and St. Gallen and the Hospital Schwyz in Switzerland, as well as the Clinic Hietzing, Vienna in Austria and the Antonius Hospital Nieuwegein in the Netherlands between May 2021 and December 2022 (estimated).

### Eligibility criteria {10}

Hospitalized patients with diagnosed COVID-19 are asked to take part in this study when the following eligibility criteria are fulfilled:

- ▪ SARS-CoV-2 infection, verified according to current in-house guidelines
- ▪ High risk for the development of ARDS:
  i.e., within 24 h before inclusion at least 2 points on an adapted EALI (early acute lung injury) score, with at least one point from the original EALI score [52, 53]
  - Original EALI score:
    - ◦ 2–6 l O_2_ supplementation to achieve a SaO_2_ > 90%: 1 point
    - ◦ > 6 l O_2_ supplementation to achieve a SaO_2_ > 90%: 2 points
    - ◦ Respiratory rate ≥ 30/min: 1 point
    - ◦ Immunosuppression: 1 point
  - Modification adapting for risk factors for ARDS in COVID-19 affected patients [54]
    - ◦ Arterial hypertension: 1 point
    - ◦ Diabetes: 1 point
    - ◦ Fever > 39 °C: 1 point
- ▪ Age ≥ 18 years
- ▪ Ability to comply with the inhalation maneuver
- ▪ Ability to understand the clinical trial and sign the informed consent

The presence of any of the following exclusion criteria will lead to exclusion of the patient:

- ▪ Mechanical ventilation or intensive care treatment within current hospitalization
- ▪ Nasal high-flow cannula or continuous positive airway pressure (cPAP) ventilation at time of inclusion
- ▪ Inability to conduct inhalation therapy
- ▪ Hemodynamic instability with requirement of vasopressor therapy
- ▪ Severe comorbidities interfering with the safe participation according to the treating physician
- ▪ Previous participation in this trial, or current participation in another interventional study
- ▪ Pregnancy
- ▪ Systemic immunosuppression for chronic underlying condition (corticosteroid treatment as part of “standard care” allowed)

“Drop-out” is defined as the patient’s withdrawal of consent at any time after inclusion into the study.

### Who will take informed consent? {26a}

After a patient is identified as a potential participant, a Good Clinical Practice (GCP)-trained physician from the study team will inform the patient personally about the trial and ask for written consent by signature on the informed consent form. Each potential participant will be informed that participation in the trial is voluntary that he/she may withdraw from the study at any time with no need of justification and that withdrawal of consent will not affect his/her subsequent medical assistance and treatment. Consent or assent from authorized surrogates is not intended, patients who are not able to understand the trial or sign the informed consent form of their own accord are excluded from participation.

### Additional consent provisions for collection and use of participant data and biological specimens {26b}

Additional collection and use of participant data and biological specimens are not intended.

### Interventions

#### Explanation for the choice of comparators {6b}

Due to the novelty of the investigated disease COVID-19, global guidelines or a clear definition of standard care are not available at the time of implementation of the study. Additionally, standard treatment strategies may change during the study period in accordance with new research findings. At each study center, there are internal written guidelines for the clinical management of hospitalized COVID-19 patients, depending on the severity of the case. At the time of implementation of the study, elements of the standard care used in the participating study centers include oxygen therapy, systemic glucocorticoids, remdesivir, tocilizumab, nutritive measures, and prevention and management of ARDS, bacterial superinfections, and sepsis, as well as thrombembolic, neurologic, cardiac, and renal complications. Since there is no clear definition of standard care to date, the only ethically justifiable comparator is the best available care, which is adapted throughout the trial according to the current state of research.

#### Intervention description {11a}

Patients in the intervention group inhale 1 ml aviptadil solution (67 μg/ml) three times a day (morning, noon, evening), while participants in the control group inhale 1 ml of NaCl 0.9% three times a day. In both groups, the respective treatment is given in addition to standard care and lasts for 10 days, or until hospital discharge, whichever occurs first. The study drug is administered with the M-neb® dose+mesh nebulizer MN-300/8 in both study groups, and each application will approximately last for 10 min.

#### Criteria for discontinuing or modifying allocated interventions {11b}

Dose or device modifications are not intended. If clinically indicated, treating doctors can stop the treatment with the study drug. Participants are given the possibility to withdraw from the study at any time. Treating physicians can independently decide about additional treatment options and concomitant medication can be re-evaluated and changed at any time of this trial. A termination of the supplementation is reported to the coordinating study center immediately.

#### Strategies to improve adherence to interventions {11c}

All participants are hospitalized and medication is administered by qualified ward personnel. Ward personnel is responsible that participating patients receive the study medication in a correct manner and each administration will be documented in the patient records. Any complication or non-adherence with the administration is reported as a note to file.

#### Relevant concomitant care permitted or prohibited during the trial {11d}

All treatments considered necessary by treating doctors are permitted and their use is recorded in the case report form. Because inhaled aviptadil does not reach the systemic circulation and is mainly metabolized in the lung, pharmacokinetic interactions with aviptadil are not expected. In order to account for potential bias, we aim to analyze data from patients, who received potentially biasing concomitant treatment (e.g., immunosuppressants or immunomodulators) in an adjusted manner (see Statistical methods for primary and secondary outcomes {20a}).

#### Provisions for ancillary and post-trial care {30}

Patients are carefully monitored until discharge from the hospital and followed-up until four weeks after inclusion. Necessary aftercare is organized by treating physicians independently of this trial. No additional specific ancillary and post-trial care is planned. Despite the efforts of the research team to mitigate risks associated with the intervention, potential small harms may occur. Any potential damage or harm to participants in connection with this trial is covered by the obligatory trial insurance.

### Outcomes {12}

The primary endpoint is time to clinical improvement up to day 28, defined as the time (in days) from randomization to the decrease of at least two levels on the WHO-suggested nine-level ordinal scale (see Table 1) [55] or alive discharge from hospital, whichever occurs first. This standardized primary endpoint allows comparison with other efficacy studies in the context of the treatment of COVID-19.

**Table 1:** WHO-Ordinal scale for clinical improvement

Patient state	Descriptor	Score
***Uninfected***	No clinical or virological evidence of infection	0
***Ambulatory***	No limitation of activities	1
	Limitation of activities	2
***Hospitalized: mild disease***	Hospitalized, no oxygen therapy	3
	Oxygen by mask or nasal prongs	4
***Hospitalized: severe disease***	Non-invasive ventilation or high-flow oxygen	5
	Intubation and mechanical ventilation	6
	Ventilation + additional organ support – pressors, RRT, ECMO	7
***Dead***	Death	8

From: WHO R&D Blueprint, Novel Coronavirus COVID-19 Therapeutic Trial Synopsis [55]

*RRT* renal replacement therapy, *ECMO* extracorporeal membrane oxygenation

Key secondary endpoints are:

- ▪ Need for mechanical ventilation, non-invasive ventilation, and intensive care during hospitalization
- ▪ Occurrence of multi organ dysfunction syndrome during hospitalization
- ▪ Number of days requiring oxygen supplementation
- ▪ Change from baseline to discharge of the following biomarkers in patients’ blood samples
  - ◦ C-reactive protein (CRP)
  - ◦ Neutrophil-lymphocyte ratio
  - ◦ Interleukin-6
  - ◦ Procalcitonin
- ▪ Change from baseline to follow-up in patient-reported dyspnea, cough, and fatigue on a visual analog scale
- ▪ Patient-reported impact on health by 12-item Short Form Survey version 2 (SF-12v2) at follow-up

Other clinical endpoints include the length of hospital stay until discharge or death (in days) and mortality rate. CRP, interleukin-6, and procalcitonin are measured at baseline, at least every seven days, and at discharge. The safety endpoints adverse events (AEs), including those leading to discontinuation of treatment, serious adverse events (SAEs), and death are also reported.

### Participant timeline {13}

**Fig. 1:**
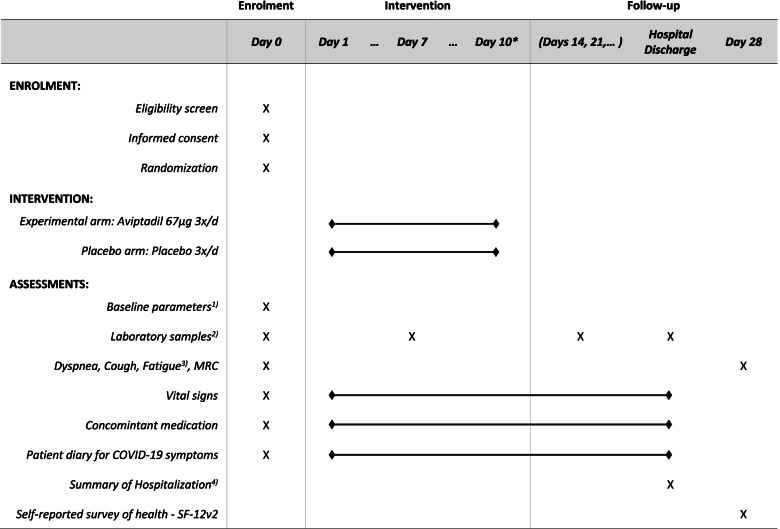
Participant timeline. Asterisk symbol (*) indicates the following: if hospital discharge occurs first, intervention is stopped at discharge. Superscript digit one (1) indicates the following: demographics, clinical status according to nine-level ordinal scale, medical history, smoking status, COVID-19 symptoms, and COVID-19 vaccination status. Superscript digit two (2) indicates the following: C-reactive protein, neutrophil-lymphocyte ratio, interleukin-6, and procalcitonin. Superscript digit three (3) indicates the following: on a visual analog scale from 0 to 10. Superscript digit four (4) indicates the following: Clinical status on nine-level ordinal scale, admission to ICU, ventilation, mortality, complications. MRC, Medical Research Council dyspnea scale; SF-12v2, 12-item Short Form Survey version 2

The participant timeline is exhibited in Fig. 1.

### Sample size {14}

Based on the effect of aviptadil in previous trials described in the introductory section, we assume that patients in the experimental group will show a 1.75-fold hazard to have a clinical improvement as compared to patients in the control group (hazard ratio (HR) = 1.75) [42, 50]. Furthermore, based on previously published results for current standard care, we assume an overall probability of 80% for reaching the primary endpoint (clinical improvement) within 28 days (*d* = 0.8) [56, 57]. Group allocation shall be divided equally ($\frac{1}{2}n={n}_1={n}_2$).

Testing for two-sided equality in a Cox proportional hazards model at a significance level of *α* = 0.05 and aimed power of 80% (*β* = 0.2), we are applying the sample-size formula in Chow as follows [58]:

$$n=\frac{{\left({z}_{\alpha /2}+{z}_{\beta}\right)}^2}{b^2\ast 0.25\ast d}=\frac{{\left(1.96+0.84\right)}^2}{{\mathit{\log}}^2(1.75)\ast 0.25\ast 0.8}\approx 125.2$$

where *b* =  *log* (*HR*) =  *log* (1.75).

Estimating a drop-out rate of 3% and considering block randomization, we aim to include 132 patients into the study.

### Recruitment {15}

All hospitalized patients at a study center who fulfill the eligibility criteria are asked to take part in this study. In order to reach the target sample size within the planned time frame, the study is geographically expanded to several centers within Europe.

### Assignment of interventions: allocation

#### Sequence generation {16a}

The group-allocating randomization code is a computer-generated sequence, using block randomization with block size of four. Each block determines assignment of four patients to the two groups, while two patients are assigned to the intervention group and two patients are assigned to the control group in a random order. Randomization is stratified by study center in order to account for differences in standard of care. Block randomization also accounts for differences in standard of care according to time elapsed since the start of the pandemic.

#### Concealment mechanism {16b}

The entire concealment process is handled in the pharmacy that produces the study drug. Identically looking vials with doses of either 3 ml aviptadil solution (67 μg/ml, experimental group) or 3 ml NaCl 0.9% (placebo group) are filled in the pharmacy in a 1:1 ratio. Kits of 10 vials of the same content (one vial per treatment day, for a maximum of 10 days) are then packed and labeled with a continuous kit number. The actual content of each kit (aviptadil or placebo) is determined by the randomization sequence described above, while the kit number represents the concealed randomization code. For each kit, the description of the actual content (aviptadil or placebo) is packed in a separate concealed envelope, with only the kit number visible from the outside. The respectively allocated kits and concealed envelopes are shipped to the coordinating study center in Liestal. From here, they are distributed to each study center and used in the order of the kit number indicated on the label. Upon inclusion of a patient, the allocated kit number is documented in the CRF. The concealed envelopes containing the description of group allocation are stored at the coordinating study center in Liestal.

#### Implementation {16c}

The entire concealment process is handled in the pharmacy that produces the study drug. Study physicians at the respective study center will enroll participants into the study and hand out the kits containing the study drug in the order of the assigned kit numbers. Group assignment is pre-defined by order of the kit numbers and the content of the respective kit.

### Assignment of interventions: blinding

#### Who will be blinded {17a}

Trial participants, investigators, treating physicians, study personnel administering the inhalation, and data analysts are blinded to group allocation. Since the study drug is filled into identically looking vials and aviptadil solution and placebo are not visually distinguishable, blinding is ensured until unblinding procedures are actively undertaken.

#### Procedure for unblinding if needed {17b}

If a treating physician of a participating patient needs to know for medical reasons (i.e., AE, SAE), if the patient is assigned to the interventional or control group, he/she can call a 24-h phone hotline to receive information about the patient’s group allocation. Unblinding is undertaken by a trained study team member, who opens only the envelope with the respective kit number.

### Data collection and management

#### Plans for assessment and collection of outcomes {18a}

All required patient data at baseline, during hospitalization, at discharge and follow-up are recorded on paper-based case report forms (CRFs). Trained study personnel at each site transfer the data into a web-based electronic case report form. A copy of the CRF used in the trial is available on reasonable request. Laboratory analyses comprise only standard parameters and are performed in the accredited in-hospital laboratories at each site. Patient dyspnea is assessed at inclusion and follow-up by means of the Medical Research Council dyspnea scale (MRC), which has been studied in a variety of respiratory conditions, including COVID-19. Patient-reported outcome is assessed at follow-up by means of the instrument SF-12v2, which is a validated survey for investigating health-related quality of life in a variety of both acute and chronic conditions, including lung diseases.

#### Plans to promote participant retention and complete follow-up {18b}

Patients participating in the study do not receive financial reimbursement. Expenses for the study medication and diagnostics performed only during this study are covered by the study budget. The 28-day follow-up is a phone call, initiated by a study team member at the respective study center. In case of withdrawal of consent or premature termination of the study, data and samples are evaluated in encrypted form until termination of the study.

#### Data management {19}

Data acquisition and entry into the web-based and password-protected electronic data capture system (SecuTrial®) are performed by trained staff at the participating study centers. Personal contact information, which is needed for follow-up phone calls, is stored separately and only accessible for the staff members executing these phone calls. Any paper documents, such as informed consent forms, are stored in locked cabinets in restricted access areas at the respective participating study centers. Electronic records are stored on a password-protected server. All records are archived for a minimum of 10 years after study termination or premature termination of the clinical trial at the respective participating study center. A data management plan documents details for the data processing and is available from the authors upon reasonable request.

#### Confidentiality {27}

All collected patient data are treated as confidential and stored and analyzed in a coded way in accordance with data protection principles. Results will be published in anonymized fashion. Direct access to source documents is permitted for purposes of monitoring, audits, and inspections. The study data and protocol shall be accessible to regulatory authorities for at least ten years after study termination.

#### Plans for collection, laboratory evaluation, and storage of biological specimens for genetic or molecular analysis in this trial/future use {33}

The trial does not involve collecting biological specimens for genetic or molecular analysis.

## Statistical methods

### Statistical methods for primary and secondary outcomes {20a}

R will be used for all statistical analyses [59]. Primary and secondary endpoints will be summarized by group using descriptive statistics (mean ± standard deviation for normally distributed data, median and interquartile range for other continuous data, absolute and relative frequencies for categorical data). A log-rank test will be used to compare Kaplan-Meier curves for the primary endpoint “time to clinical improvement,” with failure to reach clinical improvement or death before day 28 being right-censored at day 28. Sensitivity analyses will be performed with “death” and “clinical improvement” as competing risks. For potential confounders that are not fully balanced by randomization, adjusted analyses for the primary outcome may be performed using Cox regression models to estimate hazard ratios with corresponding two-sided 95% confidence intervals to compare with the respective unadjusted Cox model. The proportionality assumption for the Cox proportional hazards models will be tested by assessing Schoenfeld residuals.

Treatment effects on binary secondary endpoints will be analyzed by means of chi-squared test, or Fisher’s exact test, in case expected sub-group size is smaller than five. For discrete secondary endpoints, group differences will be assessed using a Poisson or negative binomial regression, depending on the underlying distribution. Treatment effects on patient-reported severity of symptoms will be analyzed with an ordinal logistic regression and an additional longitudinal analysis with cumulative link mixed models. Effects on longitudinal changes in infection-related biomarkers will be assessed using mixed effect linear regression and analysis of covariance. Patient-reported impact on health at day 28 will be compared between the two groups by means of Student’s *T*-test, or Mann-Whitney-*U* test, depending on normality of the data.

All statistical tests will be performed using two-sided tests at the significance level 0.05. Detailed methodology for statistical analyses of the data collected in this trial is documented in a separate statistical analysis plan (SAP). The SAP is finalized before database closure and can be obtained from the authors upon reasonable request. A summary of the SAP is provided in Additional file 1.

### Interim analyses {21b}

Since a relatively small number of patients is studied during a short study period (max. 28 days) and the treatment harm is considered to be very small due to the short treatment period, we do not consider stopping criteria nor interim analyses to assess the probability that the benefit exceeds the clinically important difference.

### Methods for additional analyses (e.g., subgroup analyses) {20b}

See Statistical methods for primary and secondary outcomes {20a}.

### Methods in analysis to handle protocol non-adherence and any statistical methods to handle missing data {20c}

The primary analysis will be done in the intention-to-treat population and safety analysis will be done on all patients who started their assigned treatment. The time to clinical improvement will be assessed after all patients will have reached day 28, with failure to reach clinical improvement or death before day 28 considered as right censored at day 28. Careful trial planning and conduct will minimize the occurrence of missing data as far as possible. In case of missing data, treating physicians are contacted with the aim to complete missing data from patients’ records. No data imputation is planned.

### Plans to give access to the full protocol, participant level-data and statistical code {31c}

Further and updated trial information can be found at www.ClinicalTrials.gov, NCT04536350. Data and materials that support this protocol, such as a detailed data management plan, CRFs and informed consent form are available from the authors on reasonable request.

## Oversight and monitoring

### Composition of the coordinating center and trial steering committee {5d}

The coordinating study center at the Cantonal Hospital Baselland, Liestal, Switzerland, consisting of the sponsor-investigator, co-investigators, study coordinators, study physicians, study nurses, and study statistician supervises and coordinates the study. This includes trial management and coordination, statistical, and economic and data management, as well as organizational support for participating study centers. It furthermore acts as a trial steering committee.

### Composition of the data monitoring committee, its role and reporting structure {21a}

The monitoring of this study is performed by the independent clinical trial unit (CTU) of the University Hospital Basel, Switzerland, in collaboration with qualified personnel of the coordinating study center. Reporting is done directly to the Investigator. The CTU is independent of the sponsor and without competing interests.

### Adverse event reporting and harms {22}

During the entire duration of the trial, all AEs and SAEs are collected, fully investigated, and documented, irrespective of whether they are related or unrelated. Participating study centers are obliged to report SAEs within 24 h to the sponsor-investigator, who, in case of death, reports to the ethics committee within 7 days. AEs and SAEs are followed up until resolution or stabilization. Adverse events will be reported in any associated relevant publication arising from this trial.

### Frequency and plans for auditing trial conduct {23}

The study is conducted in accordance with the currently approved protocol, GCP standards, and relevant regulations. Regular monitoring is performed following GCP and the trial monitoring plan. Data is evaluated for protocol compliance, integrity, and accuracy in relation to source documents. Authorities can audit this trial independently from the Sponsor-Investigator and the data monitoring committee. Study documentation and data are accessible to auditors and all involved parties must treat participant data as strictly confidential.

### Plans for communicating important protocol amendments to relevant parties (e.g., trial participants, ethical committees) {25}

Important protocol modifications are submitted to the relevant authorities (ethics commission, local drug authorities) for approval before implementation. Changes are communicated to other relevant parties (investigators, study physicians, study nurses) via E-Mail newsletters and personal phone calls.

### Dissemination plans {31a}

Our aim is to publish the results of this study in a peer-reviewed journal, without the engagement of professional writers.

## Discussion

Our described study protocol presents the design for a randomized controlled trial to investigate the efficacy and safety of inhaled aviptadil in patients hospitalized for COVID-19 at high risk for ARDS. In the context of upcoming new variants of SARS-CoV-2 and a possible future endemic state, the investigation of alternative therapy options still plays a crucial role in decreasing associated mortality and improving prognosis. Due to its unique immunomodulating properties specifically targeting the SARS-CoV-2 pathways, inhaled aviptadil may have the potential to prevent ARDS in COVID-19.

This trial is conducted in different European hospitals in order to ensure generalizability and meet the recruitment target. Patients of all adult age groups and with a wide range of co-morbidities are included into the study, allowing for a broad representation of COVID-19 patients. The primary endpoint as suggested by the WHO allows a comparison with other investigated substances for the management of COVID-19. The evaluation of the secondary endpoints will give further insight into the effects of inhaled aviptadil with regard to systemic inflammation markers, as well as patient-reported outcomes.

Due to the novelty of the disease and new research results, standard care is constantly adapted, which may lead to changes in the relative effect of aviptadil over time. However, the block-randomization with a small block-size ensures that this circumstance will not bias the results. Furthermore, with COVID-19 being the research focus of many current projects, which are “competing” for participating patients, recruitment may become difficult. This challenge is addressed by increasing the number of collaborating centers and assuring, that no competing projects are recruiting at the involved sites.

To date, there are no published data about the administration of inhaled aviptadil in COVID-19. Because of the promising properties of aviptadil, there is an urgent need to close this research gap with a well-designed randomized controlled trial. If the results of this study show that inhaled aviptadil is effective and safe, they may ultimately lead to the introduction of a new substance for the management of COVID-19.

## Trial status

The first patient was enrolled into this study on May 18, 2021. The study is currently ongoing with active recruitment under protocol version 8, dated June 30, 2022. Recruitment is anticipated to be completed in December 2022.

## Supplementary Information

**Additional file 1.** Summary of statistical analysis plan.

## Data Availability

Data and materials that support this protocol, such as a detailed data management plan, CRFs, and informed consent form are available from the authors on reasonable request. Following completion of the trial, anonymized datasets and statistical code used in this study will be available from the authors on reasonable request.

## Abbreviations

AE: Adverse event
ARDS: Acute respiratory distress syndrome
ATII: Alveolar type-II
cPAP: Continuous positive airway pressure
CRF: Case report form
CRP: C-reactive protein
CTU: Clinical trial unit
DMC: Data monitoring committee
EALI: Early acute lung injury
EKNZ: Ethics Committee for the Region of Northwestern and Central Switzerland
EKOS: Ethics Commission for Eastern Switzerland
GCP: Good Clinical Practice
ICU: Intensive care unit
SAE: Serious adverse event
SAP: Statistical analysis plan
SF-12v2: 12-item Short Form survey, version 2
VIP: Vasoactive intestinal polypeptide
WHO: World Health Organization
