# Supplementary material for: Inhaled aviptadil for the possible treatment of COVID-19 in patients at high risk for ARDS: study protocol for a randomized, placebo-controlled, and multicenter trial
Source: Trials. 2022 Sep 20;23:790. doi: 10.1186/s13063-022-06723-w (PMC9486780; doi:10.1186/s13063-022-06723-w)
Supplement: Supplementary file 1 — Additional file 1. Summary of statistical analysis plan. [file 13063_2022_6723_MOESM1_ESM.pdf]

Additional file 1 : Summary of Statistical Analysis Plan
Inhaled Aviptadil for the possible treatment of COVID-19 in patients at high risk for ARDS

Objective	Outcome(s)		Hypothesis	Method of Analysis
	Name	Variable Type		
**Primary Objective**				
Compare time to recovery	Time to clinical improvement	Continuous	Inhalation of Aviptadil for 10 days in addition to standard care will decrease the time to clinical improvement when compared to standard care.	Kaplan Meier / log-rank / Cox-proportional hazards
**Secondary Objectives**				
Compare risk of needing mechanical ventilation during hospitalization	Mechanical ventilation, Number of days needing mechanical ventilation	Binary, Count	Inhalation of Aviptadil for 10 days in addition to standard care will decrease the risk of needing mechanical ventilation when compared to standard care.	Chi-Squared / Fisher's exact test*, Poisson / negative binomial regression**
Compare risk of needing non-invasive ventilation during hospitalization	Non-invasive ventilation, Number of days needing non-invasive ventilation	Binary, Count	Inhalation of Aviptadil for 10 days in addition to standard care will decrease risk of needing non-invasive ventilation when compared to standard care.	Chi-Squared / Fisher's exact test*, Poisson / negative binomial regression**
Compare risk of multi-organ dysfunction syndrome during hospitalization	Multi-organ dysfunction syndrome	Binary	Inhalation of Aviptadil for 10 days in addition to standard care will decrease risk of multi-organ dysfunction syndrome when compared to standard care.	Chi-Squared / Fisher's exact test*
Compare number of days requiring oxygen supplementation	Number of days requiring oxygen	Count	Inhalation of Aviptadil for 10 days in addition to standard care will decrease the number of days requiring oxygen when compared to standard care.	Poisson / negative binomial regression**
Compare mortality at 28 days	Mortality	Binary	Inhalation of Aviptadil for 10 days in addition to standard care will decrease mortality when compared to standard care.	Chi-Squared / Fisher's exact test*
Compare risk of needing intensive care within 28 days	Intensive care, Number of days needing intensive care	Binary, Count	Inhalation of Aviptadil for 10 days in addition to standard care will decrease the risk of needing intensive care when compared to standard care.	Chi-Squared / Fisher's exact test*, Poisson / negative binomial regression**
Compare length of hospital stay	Length of Hospital Stay (Days)	Count	Inhalation of Aviptadil for 10days in addition to standard care will decrease length of hospital stay when compared to standard care.	Poisson / negative binomial regression**
Compare patient-reported severity of symptoms at 28 days	MRC, Severity of Cough, Severity of Fatigue, Severity of Dyspnea	Ordinal	Inhalation of Aviptadil for 10 days in addition to standard care will decrease patient-reported severity of symptoms when compared to standard care.	Ordinal logistic regression
Compare change in patient-reported severity of symptoms from baseline to day 28	Change in: MRC, Severity of Cough, Severity of Fatigue, Severity of Dyspnea	Ordinal	Inhalation of Aviptadil for 10 days in addition to standard care will result in more frequent/earlier recovery from symptoms suffered at baseline when compared to standard care.	Cumulative Link Mixed Models with one random term (subject) and fixed effect with group by time interaction
Compare patient-reported impact on health at 28 days	SF-12v2 Physical component, SF-12v2 Mental component	Continuous	Inhalation of Aviptadil for 10 days in addition to standard care will improve patient-reported impact on health at 28 days when compared to standard care.	T-test / Mann-Whitney-U-Test**
Compare change in inflammatory biomarkers from baseline to discharge	Change in: CRP, neutrophil-lymphocyte-ratio, interleukin-6, procalcitonin	Continuous	Inhalation of Aviptadil for 10 days in addition to standard care will result in a stronger decrease of the listed inflammatory biomarkers when compared to standard care.	ANCOVA (adjusted for baseline value) / Mixed effect linear regression with one random term (subject) and fixed effect with group by time interaction

Additional file 1 : Summary of Statistical Analysis Plan
Inhaled Aviptadil for the possible treatment of COVID-19 in patients at high risk for ARDS

Objective	Outcome(s)		Hypothesis	Method of Analysis
	**Name**	**Variable Type**		
**Subgroup Analyses**				
not planned				
**Safety Analysis**				
Asess any potential harm of inhaled Aviptadil	Number of adverse events	Count	There will be no difference in the number of adverse events, irrespective of group allocation.	Poisson / negative binomial regression**
**Sensitivity Analyses**				
Competing risks (death and recovery)	Time to clinical improvement	Continuous	We do not expect the association between inhalation of Aviptadil and time to clinical improvement to change substantially once we take into account the competing "risk" of death.	Competing risk analysis
Trial site (center-effects)	Time to clinical improvement	Continuous	We do not expect substantial changes to the results when adjusting for potential center-effects.	Cox-proportional hazards (adjusted for study center)
Potential confounders not fully balanced by randomization at baseline	Time to clinical improvement	Continuous	Results will remain robust after adjusting for the following baseline imbalances: age, sex, oxygen-requirements at baseline, presence of comorbidity arterial hypertension, presence of comorbidity diabetes mellitus	Cox-proportional hazards (adjusted for respective potential confounder)
Potential bias through concomitant medication	Time to clinical improvement	Continuous	Results will remain robust after adjusting for administration of the following concomitant medication: corticosteroids, remdesivir, tocilizumab, baricitinib	Cox-proportional hazards (adjusted for respective concomitant medication)

* in case expected number of absence / presence of outcome per randomized group contains less than five participants, Fisher's exact test will be used
** depending on underlying distribution
